# Incident and Emergency Medical Services Management from a Regional Perspective 

**DOI:** 10.3390/ijerph9072266

**Published:** 2012-06-26

**Authors:** Virginia P. Sisiopiku, Ozge Cavusoglu

**Affiliations:** 1 Department of Civil, Construction, and Environmental Engineering, University of Alabama at Birmingham, 1075 13th Street South, Birmingham, AL 35294, USA; 2 Regional Planning Commission of Greater Birmingham, 1731 1st Avenue North, Birmingham, AL 35203, USA; Email: ocavusoglu@rpcgb.org

**Keywords:** traffic incident management, EMS response, DTA, VISTA, Birmingham

## Abstract

Traffic crashes and other emergencies have impacts on traffic operations in transportation networks, often resulting in non-recurring congestion. Congestion, in turn, may impede the ability of Emergency Medical Services (EMS) to provide timely response to those in need of medical attention. The work in this paper investigated the impact of incidents of varying severity and duration on transportation network performance in the Birmingham (AL, USA) area. The intensity and extent of the impact over space and time were assessed on the basis of average speeds. The analysis of incident scenarios was performed using the Visual Interactive System for Transport Algorithms (VISTA) platform. Moreover, first responders’ travel times to the scene of the incident were collected to identify best units for responding, in an effort to improve current dispatching practices. Finally, a secondary incident on the EMS to the hospital was considered to further demonstrate the superiority of Dynamic Traffic Assignment (DTA) over traditional static assignment methods in capturing dynamically changing traffic conditions. The study findings are expected to benefit local transportation planners, traffic engineers, emergency responders, and policy makers by allowing them to assess various response strategies to major incidents and emergencies and select the ones that minimize their potential impacts.

## 1. Introduction

Traffic incidents and other emergencies are highly likely to impact transportation network performance when they occur. Increase in delays, reduction in traveling speeds and formation of queues are commonly observed upstream of incident sites. Reduction in supply associated with lane closures and/or increase in demand as a result of traffic bottlenecks may also impede the ability of first responders to reach the scene of the incident and transport victims to emergency rooms in a rapid and efficient manner.

Understanding the impacts of incidents on traffic operations is very important as it enables authorities to: (a) better manage the traffic so that it minimizes undesirable impacts on traffic operations and (b) manage effectively the flow of first responders to/from the site of the incident. Lessons learned from past experiences confirm that in case of incidents or emergencies, effective real-time traffic management is essential to avoid deterioration of traffic conditions [1]. Therefore, in support of incident management, there is a need for models which can capture the fast changing dynamic traffic conditions taking into consideration traffic management measures implemented to meet the management objectives stated above and potential infrastructure failures. It should be also stated that in the case of emergencies, drivers behaviors are often altered, which makes existing models not directly applicable. In order to address the above stated considerations, simulation-based Dynamic Traffic Assignment (DTA) models can be utilized to estimate time-varying network conditions by capturing fast changing dynamic traffic flows and route choice behavior [2].

### Study Objectives

The objective of this study was to demonstrate the use of simulation-based DTA models as a tool for evaluating the impacts of incidents of varying duration and intensity on traffic operations. The work focused on the development of a comprehensive regional model of the Birmingham (AL, USA) region in the VISTA platform that can be used as a training and evaluation test bed. Special attention was placed on optimizing decision-making in responding to traffic incidents in the Birmingham region. 

## 2. Methodology

### 2.1. Study Approach

In this research, a simulation study was performed to examine the impacts of major incidents and determine the best response time of emergency units. For that reason a regional model of the Birmingham network was developed. More specifically, the study employed a simulation-based DTA platform to model normal operations (base case) and incident conditions. In the incident scenarios considered in this study, the number of lanes closed and the duration of lane closures was varied to represent incidents of various levels of severity. Furthermore, the selection of vehicle optimal travel paths took under consideration information availability and network familiarity. In the presence of an incident, drivers were expected to stay at their original paths if they had no information about potential diversion (simulation scenarios). On the other hand, driver knowledge of the incident and familiarity with diversion options would allow them to seek new, improved paths in order to avoid delays due to the incident (DTA scenarios). Detailed information about the simulation model selected, study site used, and the simulation scenarios considered is presented in the following paragraphs. 

### 2.2. Simulation-Based DTA Model Selection

The simulation model capabilities required to meet the objectives of this paper include the ability to simulate transportation network operations and driver behaviors under normal and incident conditions, the ability to determine network-wide, corridor-wide measures of performance such as delay, travel time and speed, and the ability to track individual vehicles in the network so that one can track emergency vehicles and obtain information about their response time of to/from the incident site. Furthermore, the model should be capable of simulating networks that are large enough to allow observation of the direct effects of incidents and the pre-planned management strategies, but also the indirectly impacted areas. 

A detailed review of the model approaches, capabilities, and limitations, along with the availability of models and other resources, led to the selection of VISTA as tool of choice for this study. VISTA utilizes a mesoscopic simulator (RouteSim) and a Dynamic Traffic Assignment (DTA) routine to emulate the behavior of individual drivers and how they distribute themselves into the transportation network. RouteSim is based on an extension of Daganzo’s cell transmission model introduced by Ziliaskopoulos and Lee [3]. The road is divided into small cells where the cells are adjustable in length; bigger cells are used for a mid-section of a long highway segment, and smaller cells are used for intersections and interchanges. Vehicles are considered to be moving from one cell to another in platoons. The simulator keeps track of the flow in each cell and for every time step, calculates the number of vehicles that are transmitted between adjacent cells. The model enables the study of incidents’ impacts as it generates spatial-temporal traffic flows for all origin-destination trips loaded into the network. Furthermore, the VISTA system can emulate the routes that the emergency vehicles should follow in order to arrive at the scene of an incident given the prevailing traffic conditions.

### 2.3. Development of Simulation Model for the Birmingham Region

**Figure 1:**
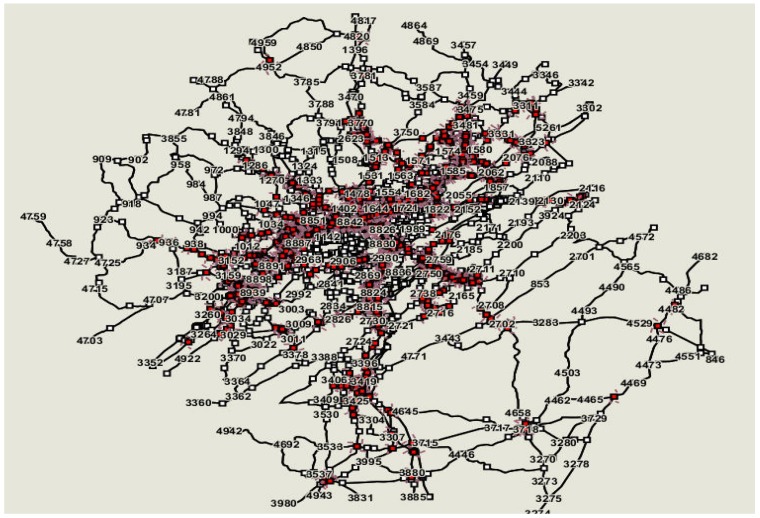
Birmingham network coded in VISTA.

The study network of the Birmingham region was built in VISTA using background geometric and annual average daily traffic (AADT) volume data from the TRANPLAN (TRANsportation PLANning) model provided by the Regional Planning Commission of Greater Birmingham (RPCGB). More than 1.8 million trips with more than 35 million route options were simulated for the afternoon peak period starting from 4:00 to 7:00 PM. The coded network consists of about 11,500 links including major freeways (e.g., I-65, I-459 and, I-20/59) and several arterial facilities serving the Birmingham region. Figure 1 shows the Birmingham network coded in VISTA. For the purpose of the study, existing fire and police stations as well as medical facility locations were geo-coded and included in the simulation model. 

As part of the model development, a model calibration process was included to ensure that the model replicates closely real conditions. Traffic volume data collected from loop detectors by ALDOT were used for the VISTA model calibration and refinement (Table 1). 

**Table 1:** Traffic volume data.

DATA CATEGORY	DATA
**Counter IDIN**	37–95
**Station **	95
**County**	37
**City**	35
**Route**	65
**Mile point**	250.08
**AADT 2009**	**115,150**
**AADT 2008 **	113,900
**AADT 2007 **	118,520
**AADT 2006 **	117,930
**AADT 2005 **	117,800
**AADT 2004 **	115,060
**AADT 2003 **	113,300
**AADT 2002 **	109,720
**AADT 2001 **	110,210
**K **	10
**D **	65
**TDHV **	8
**TADT **	11
**Heavy **	70
**Functional Class **	11

Moreover, a series of field travel time studies took place that enabled the comparison of actual (field) travel times to those generated by the prototype model developed in this study. The travel time data were collected along the I-65 corridor (between Valleydale Rd. and I-20/59) for three weeks in the Fall of 2009, on Tuesdays, Wednesdays and Fridays during morning and afternoon rush hours. Table 2 shows a sample calculation of delay time data for the selected exits on I-65 corridor during AM peak and Table 3 presents a comparison of field delay time and VISTA delay time data on the same selected exits. Overall, a close agreement between field observations and simulated travel times was observed.

**Table 2:** Sample delay time calculations.

I-65 North Bound AM				
I-65 Exits	Field Travel Time Data (min:sec.msec)	Field Travel Time (sec)	Ideal Travel Time (sec) *	Delay Time (sec)
247–250	03:37.3	926	660	266
250–252	03:57.5			
252–254	02:50.0			
254–255	01:50.5			
255–256A	01:12.0			
256A–258	02:00.4			
258–259	01:59.7	264	180	84
259–260	00:54.0			
260–261A	01:31.6			
TTI = Posted Speed Limit */Travel Speed (* Posted Speed Limit is 60 mph on I-65 corridor)				

**Table 3:** Sample Delay Time Comparisons for Model Calibration.

I-65 North Bound AM				
I-65 Exits	Calculated Delay Time (sec)	Calculated Delay Time (min)	Vista Links correspond to I-65 Exits	Vista Delay Time (min)
247–250	266	4.43	9090 (247–250)	4.62
250–252			9038, 9046 (250–252)	
252–254			6913, 12749 (252–254)	
254–255			6995, 7003 (254–255)	
255–256A			12132, 12131 (255–256)	
256A–258			7069, 7063 (256–258)	
258–259	84	1.40	2402, 2419 (258–259)	1.41
259–260			4561, 10307, 12299, 5629 (259–260)	
260–261A			5797, 5994 (260–261A)	

### 2.4. Study Site

Analysis of historical crash data from the CARE database took place to identify a good candidate location for the generation of a traffic incident within the study area. The CARE database is a comprehensive crash database for the state of Alabama that is maintained by the University of Alabama, Tuscaloosa. Records show that more than 200 traffic incidents occurred in 2008 at the I-65/I-459 junction, with a clearance time ranging between 10 and over 180 minutes. Using this input a primary incident for the incident scenarios was created on the southbound I-65 highway just upstream of the I-459 junction (US-31/Montgomery Hwy between exits 252 and 250). In VISTA, the incident was modeled on link #12,750, which is an 8,500 foot-long 4-lane interstate segment with a capacity of 2,300 vphpl, and a free flow speed of 65 mph. Moreover, historical records show that PM peak hours experience the highest incident rates at this junction, thus, afternoon peak was used as the analysis period in this study.

While the entire regional network was considered in the simulation runs, in depth analysis of network performance was deemed more appropriate in the vicinity of the incident. Thus a 13-mile length segment of I-65 was chosen as the study corridor for which results were obtained and analysed in greater detail. The segment was divided into 12 sub segments, which were defined based on the actual highway exit points. The details are provided in Table 4. Accordingly, three main incident scenarios were designed for the Birmingham network to analyze the impacts of incidents of varying severity and study emergency response. For this purpose, six different incident severity levels were defined by varying the incident duration and the number of lanes closed due to the incident. 

**Table 4:** I-65 study corridor segments.

	Segments	From Exit on I-65	To Exit on I-65
**1**	6th Ave. N	Exit 260	Exit 260B
**2**	3rd Ave. N	Exit 260B	Exit 259B
**3**	4th Ave. S	Exit 259B	Exit 259A
**4**	6th Ave. S	Exit 259A	Exit 259
**5**	AL-149/University Blvd.	Exit 259	Exit 258
**6**	Green Springs Ave.	Exit 258	Exit 256
**7**	Oxmoor Rd.	Exit 256	Exit 255
**8**	Lakeshore Dr.	Exit 255	Exit 254
**9**	Alford Ave.	Exit 254	Exit 252
**10**	US-31/Montgomery Hwy	Exit 252	Exit 250
**11**	I-459	Exit 250	Exit 247
**12**	CR-17/Valleydale Rd.	Exit 247	Exit 246

- *Scenario 1 (S1)* described the network operations under non-incident conditions and provided the baseline for comparisons.
- In *Scenario 2 (S2)* a sensitivity analysis took place to illustrate the impact of the incident severity on travel times, delays, and response times of emergency units. In *Scenario 2* the incident duration varied in 60 minute increments under one-to-two full lane blockage conditions and the relative changes in model response were observed. The scenario assumed the occurrence of the primary traffic incident on southbound I-65 at the junction of I-65 and I-459 starting at 4:00 PM. Scenarios S2-11 and S2-12 assumed an incident lasting for 1 h and closing 1 and 2 lanes respectively. Scenarios S2-21 and S2-22 assumed an incident-duration of 2 hours with 1 and 2 lane reduction respectively. Finally, in scenarios S2-31 and S2-32 the simulate incident persisted for 3 hours with 1 or 2 lane closures respectively.
- In *Scenario 3 (S3)*, the primary incident conditions were assumed to be the same as in S2-22 (*i.e.*, two lanes closed for 2 hours); however, a secondary incident was introduced along the route of the best available responding EMS unit to the main hospital. The secondary incident was assumed to result in a two-lane blockage at the I-65/Lakeshore Dr. on I-65 northbound. The blockage started at 4:20 PM and lasted for at least 30 minutes. The objective of this scenario was to study the impact of the secondary accident on travel times of emergency units heading toward a hospital.

Two sets of runs were performed for each incident scenario introduced above. The first set (denoted by S) assumed that the drivers had no information about the incident presence. Under this assumption the Simulation Module of VISTA was run for the study scenarios (*i.e.*, S2-11S, S2-12S, S2-21S, S2-22S, S2-31S, S2-32S, and S3-22S). In doing so, the RouteSim simulator utilized the optimal paths from the base case and determined the impact of the incident on the same Origin-Destination (OD) paths, since the drivers remained in these same paths due to lack of information related to incident occurrence.

The second set of runs (denoted by D) assumed that drivers knew about the incident and they redistribute themselves in the network according to the DTA principles in order to re-optimize their paths as needed, given the presence of the incident. During the VISTA Dynamic Traffic Assignment/Dynamic User Equilibrium (DTA/DUE) procedure, the RouteSim initially assigned the vehicles to the free flow shortest paths. The link travel times resulting from that assignment pattern were then used to calculate a new set of shortest paths, and the simulation is repeated with vehicles assigned to a combination of the previously calculated path set. Iterations continue between the mesoscopic simulation and vehicle assignment modules until a user-specified convergence criterion is met. In this study the convergence criterion was set to a 4% gap, in order to increase the accuracy and confidence in the model findings. 

Using its DTA/DUE module, VISTA recalculated all vehicle paths and re-optimize routes given the incident presence for the scenarios considered above (*i.e.*, S2-11D, S2-12D, S2-21D, S2-22D, S2-31D, S2-32D, and S3-22D). The gaps achieved in the various scenarios considered varied from 3.20% to 3.89%. 

The comparison between the results of a simulation only and DTA/DUE optimization scenario allows the analyst to study the effect of information provision on network performance. Table 5 summarizes the study scenarios and the corresponding VISTA module used to determine the network performance.

## 3. Results

VISTA provides general reports summarizing network performance measures such as travel times, vehicle throughput, and speeds. Moreover, queries can be executed to obtain detailed information about facilities of interest. Such queries where utilized to obtain speeds for twelve I-65 links located upstream and downstream of the incident location. To allow evaluation of incident impacts over space and time, both average speeds (*i.e.*, link speeds over the 4 hr simulation period), and 15-min speeds for each link were obtained. Summary results are reported and compared in the sections that follow whereas detailed results are available in Sisiopiku *et al*. [4]. 

**Table 5:** Case study scenarios.

Scenario	Name	Incident Duration (h)	Lane Blockage (# of lanes)	Information Provision	VISTA Module
Scenario 1-Base Case	S1	0	0	-	DTA/DUE
Scenario 2-Primary Incident only	S2-11S	1	1	no	Simulation
	S2-12S	1	2	no	Simulation
	S2-21S	2	1	no	Simulation
	S2-22S	2	2	no	Simulation
	S2-31S	3	1	no	Simulation
	S2-32S	3	2	no	Simulation
	S2-11D	1	1	yes	DTA/DUE
	S2-12D	1	2	yes	DTA/DUE
	S2-21D	2	1	yes	DTA/DUE
	S2-22D	2	2	yes	DTA/DUE
	S2-31D	3	1	yes	DTA/DUE
	S2-32D	3	2	yes	DTA/DUE
Scenario 3-Secondary Incident	S3-22S	0.5	2	no	Simulation
	S3-22D	0.5	2	yes	DTA/DUE

### 3.1. Primary Incident Results—Without Information Provision—Scenario 2-S

Scenario S2-S Summary Results

Table 6 summarizes the average speed results for scenario 2-S. The highlighted entries show speeds on links that were affected by the incident presence as evidenced by the changes in average speeds. 

**Table 6:** Comparison of average speed results without information provision (mph).

Segments	Base–S1	S2-11S	S2-12S	S2-21S	S2-22S	S2-31S	S2-32S
6th Ave./Exit 260	63.01	63.01	63.01	63.01	63.01	63.01	63.01
3rd Ave./Exit 260B	60.00	60.00	60.00	60.00	60.00	60.00	60.00
4th Ave./Exit 259B	51.02	51.02	51.02	51.02	51.02	51.02	51.02
6th Ave./Exit 259A	42.02	42.02	42.02	42.02	42.02	42.02	42.02
University Blvd./Exit 259	66.00	66.00	66.00	66.00	66.00	66.00	66.00
Green Springs Ave./Exit 258	62.13	62.13	**53.58**	62.13	**38.71**	62.13	**38.71**
Oxmoor Rd./Exit 256	54.55	54.55	**28.65**	54.55	**18.29**	54.55	**18.22**
Lakeshore Dr./Exit 255	61.91	**60.58**	**22.00**	**59.23**	**15.51**	**59.23**	**14.70**
Alford Ave./Exit 254	55.87	**57.68**	**18.96**	**57.41**	**14.28**	**57.41**	**12.37**
US-31/Exit 252 (incident)	61.76	**60.91**	**51.83**	**60.71**	**45.45**	**60.71**	**40.47**
I-459/Exit 250	63.46	63.46	63.46	63.46	63.46	63.46	63.46
Valleydale Rd./Exit 247	63.23	63.23	63.23	63.23	63.23	63.23	63.23
**Total**	**705.0**	**704.6**	**583.8**	**702.8**	**541.0**	**702.8**	**533.2**

As the results demonstrate, the extent and intensity of speed reduction is greatly associated with available capacity (*i.e.*, number of lanes that remain open). It is clear that given the existing demand along the corridor of interest during the study period, the facility has enough reserve capacity to absorb one lane drop lasting from 1 to 3 hours without any noticeable change in performance (S2-11S, S2-21S, and S2-31S). However, when a second lane is closed, significant speed reductions were observed on links located upstream of the incident, as compared to the base case. The impact of the incident on traffic operations extended over five links (nearly 6 miles) and intensified when the 2 lane closure lasted for 2 hours (S2-22S) instead of one (S2-12S). However, further extension of the incident duration (*i.e.*, 3 hours in S2-32S) had only minor incremental effects on speed reduction compared to S2-22S. Table 7 summarizes changes in delays observed when compared to the base line from all simulation runs assuming incident presence and no information provision. 

**Table 7:** Comparison of delay results without information provision (min).

Segments	Delay Difference Compared to Base Case (min)					
	S2-11S	S2-12S	S2-21S	S2-22S	S2-31S	S2-32S
6th Ave./Exit 260	0.00	0.00	0.00	0.00	0.00	0.00
3rd Ave./Exit 260B	0.00	0.00	0.00	0.00	0.00	0.00
4th Ave./Exit 259B	0.00	0.00	0.00	0.00	0.00	0.00
6th Ave./Exit 259A	0.00	0.00	0.00	0.00	0.00	0.00
University Blvd./Exit 259	0.00	0.00	0.00	0.00	0.00	0.00
Green Springs Ave./Exit 258	0.00	**0.22**	0.00	**0.85**	0.00	**0.85**
Oxmoor Rd./Exit 256	0.00	**0.82**	0.00	**1.79**	0.00	**1.80**
Lakeshore Dr./Exit 255	**0.05**	**2.78**	**0.09**	**4.57**	**0.09**	**4.91**
Alford Ave./Exit 254	**0.08**	**3.55**	**0.09**	**5.24**	**0.09**	**6.30**
US-31/Exit 252 (incident)	**0.02**	**0.33**	**0.03**	**0.61**	**0.03**	**0.89**
I-459/Exit 250	0.00	0.00	0.00	0.00	0.00	0.00
Valleydale Rd./Exit 247	0.00	0.00	0.00	0.00	0.00	0.00

The changes in the delay results are consistent with those observed when reviewing the speed results. For a driver that traverses the entire study segment (*i.e.*, from 6th Ave./Exit 260 to Valleydale Rd.), a one lane closure does not introduce any measurable delays (S2-11S, S2-21S, S2-31S). On the contrary, an hour long two lane closure due to an incident (S2-12S) would introduce 7.7 min of extra delay to an average driver that traverses the entire study segment, in addition to the regular recurrent delay that he experiences in the base case.

When the 2-lane closure persists for 2 hours (S2-22S), the average incident-induced delay increases further to 13.06 min (14.75 min for a 2-lane, 3-hr closure in S2-32S). This is significant as it is nearly doubles the average corridor travel time compared to free flow conditions. 

Last but not least, the VISTA model results provided information about expected emergency responders travel times to the incident location given prevailing traffic conditions. Such information is very valuable for dispatching purposes as at times the emergency response unit that appears closer to an incident location may take longer than an alternate unit to arrive at the scene due to congestion.

As an example, for scenario S2-22S, among 227 emergency responder units considered, a responder unit from Hoover Fire Department—Station 1 was found to be the best one to be dispatched at the site since it could reach the incident scene within 210 seconds, or 3.5 min. The best police unit to be dispatched to the site was from Hoover Police Department with an expected travel time to the scene of 306 seconds (just over 5 min). Finally, the best EMS vehicle to be dispatched to the scene was from Galleria Woods Skilled Nursing Facility with a response time of 564 seconds (or 9.4 min). 

As Table 8 shows, due to incident-induced congestion, the best responding units have longer response times to the incident compared to travel times from their position to the incident under normal conditions (base). When such differences are large they may even lead to the selection of an alternate unit with an expected shorter response time as in the case of EMS unit dispatching in S2-22S. Based on the position of the vehicle and expected travel time to the incident location under normal operations, a dispatcher would have selected to dispatch an EMS unit from Baptist Health Center-1. However, as the study analysis indicates, when considering the incident impact on traffic conditions, the best EMS unit to be dispatched is actually one originating from Galleria Woods Skilled Nursing Facility, instead. As far as fire and police dispatching units are concerned, the same units (*i.e.*, Hoover Fire Department and Hoover Police Department) will be selected in both cases.

**Table 8:** First emergency responders arrival times and travel times.

Scenarios	Emergency Responder	Arrival Time (PM)	Travel Time sec (min)	Distance Traveled (mile)
**Base Case**	Hoover Fire Department (Station 1)	4:08:18	198 (3.3)	2.81
	Hoover Police Department	4:09:06	246 (4.1)	3.50
	Baptist Health Center–1	4:11:30	390 (6.5)	6.42
**S2-22S**	Hoover Fire Department (Station 1)	4:08:30	210 (3.5)	2.81
	Hoover Police Dept	4:10:06	306 (5.1)	3.50
	Galleria Woods Skilled Nursing Facility	4:14:24	564 (9.4)	5.88

Moreover, queries in VISTA can identify the best routes to the incident site and from the incident site to the hospital location. These details were considered in Scenario 3, the results of which are presented in a following section.

### 3.2. Primary Incident Results—With Information Provision—Scenario 2-D

The following paragraphs summarise the parametric analysis findings for incident scenario 2 assuming that the travellers have knowledge of the incident and thus they re-evaluate their original routes and select optimal paths that minimize their user cost (*i.e.*, delay, travel time *etc*.) under present (*i.e.*, incident) traffic conditions. To analyze this route choice driver behavior, the VISTA DTA/DUE Module was employed along with the RouteSim simulator.

The summary results from the parametric analysis performed are displayed in Table 9 while a complete set of results for all six S2-D scenarios (similar to the ones presented for the S2-S scenarios) is available in Sisiopiku *et al*. [4].

**Table 9:** Comparison of average speed results with information provision (mph).

Segments	Base-S1	S2-11D	S2-12D	S2-21D	S2-22D	S2-31D	S2-32D
6th Ave./Exit 260	63.01	63.01	63.01	63.01	63.01	63.01	63.01
3rd Ave./Exit 260B	60.00	60.00	60.00	60.00	60.00	60.00	60.00
4th Ave./Exit 259B	51.02	51.02	51.02	51.02	51.02	51.02	51.02
6th Ave./Exit 259A	42.02	42.02	42.02	42.02	42.02	42.02	42.02
University Blvd./Exit 259	66.00	66.00	66.00	66.00	66.00	66.00	66.00
Green Springs Ave./Exit 258	62.13	62.13	62.13	62.13	62.13	62.13	62.13
Oxmoor Rd./Exit 256	54.55	54.67	54.67	54.67	54.67	54.67	54.67
Lakeshore Dr./Exit 255	61.91	62.80	**55.86**	**62.80**	**62.80**	**62.71**	**62.80**
Alford Ave./Exit 254	55.87	59.97	**22.16**	**60.70**	**28.76**	**60.26**	**21.80**
US-31/Exit 252	61.76	61.12	**49.21**	**61.19**	**46.76**	**60.57**	**45.11**
I-459/Exit 250	63.46	63.46	63.46	63.46	63.46	63.46	63.46
Valleydale Rd./Exit 247	63.23	63.23	63.23	63.23	63.23	63.23	63.23
**Total**	**705.0**	**709.4**	**652.8**	**710.2**	**663.9**	**709.1**	**655.3**

Scenario S2-D Summary Results

Table 9 displays the average speed results for the S2-D scenarios, *i.e.*, scenarios considering the impact of incident information dissemination on network performance. As stated earlier, Scenarios S2-11D, S2-21D, and S2-31D assumed 1 lane closure for 1, 2, 3 hours respectively and Scenarios S2-12D, S2-22D, and S2-32D considered 2 lane closures for 1, 2, 3 hours respectively. The highlighted entries show speeds on links that were affected by the incident presence.

As shown in Table 9, in all cases considered, speed reductions above 25% due to the incident occur only on one link (*i.e.*, Alford Ave.) located directly upstream of the incident link (US-31) compared to 4 links in the corresponding S2-S scenarios (Table 6).

Similarly to the S2-S scenarios the impact of the number of lanes closed on performance was far more pronounced than that of the duration of the lane closure. Under the 1 lane closure scenarios (S2-11D, S2-21D, and S2-31D), the link-by-link average speeds are, in fact, fairly similar to those obtained under the base line non-incident conditions (Scenario S1). On the other hand, the 2 lane closure led to significant reductions in average speeds along Alford Ave. ranging from 48.5% to 61% (as compared with the baseline speeds), but still lower than their counterparts in the S2-S case that ranged from 66% to 77.9%. As expected scenario S2-32D showed the lowest speeds on the affected links amongst all scenarios considered but results were close to those observed under the other 2 lane closure scenarios (S2-12D and S2-22D).

**Figure 2:**
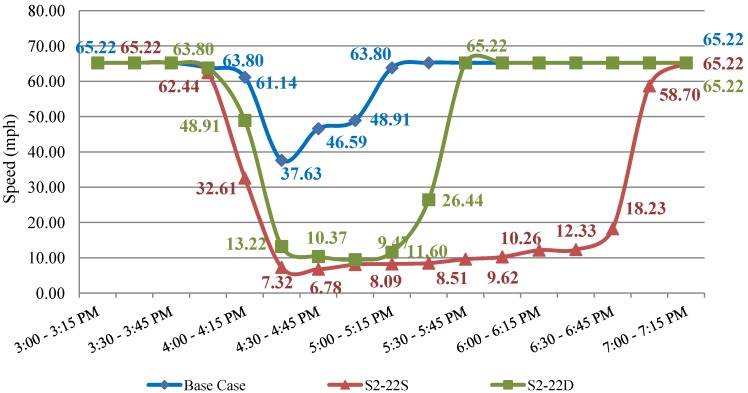
Comparison of base case, S2-22S and S2-22D: Alford Ave.

The average speed comparisons provided above can be used to obtain an overall picture of the differences between recurrent speeds and speeds under incident conditions with and without traveler information availability. Detailed comparisons between base case (S1), and all S2-S and S2-D scenarios on a link-by-link basis and over a 15-min aggregation time interval were also performed and the results are available in the [4]. For demonstration purposes, a sample such graph is provided in Figure 2. The graph compares speeds on the Alford Ave. link located immediately upstream of the incident link under a. Base Case, b. S2-22S and c. S2-22D conditions (*i.e.*, two lane closures for two hours without and with traveler information provisions).

A number of valuable findings can be extracted from close observation of Figure 2. From the base case data one can see that the link starts to experience recurrent congestion around 4:00 PM with a recovery time of around 5:00 PM. The peak 15-min period occurs at 4:15–4:30 during which the speed drops to 37.5 mph, or almost half the free flow speed. The introduction of the incident in S2-22S results in a crawling speed of 7.32 mph during the same 15-min period. Clearly the link experiences breakdown conditions which remain present for three full hours (4:00 PM to 7:00 PM). 

When drivers are aware of the incident and some divert to alternate paths (S2-22D) the impact of the incident on link operation is far more tolerable with the first signs of recovery occurring around 5:15 PM and the full recovery taking place at 5:30 PM, or 1.5 hours earlier compared to S2-22S. Similar conclusions can be derived by observation of changes in delays due to the incident presence, given availability of incident-related information. Table 10 summarizes the results. 

**Table 10:** Comparison of delay results with information provision (min).

Segments	Delay Difference Compared to Base Case (min)					
	S2-11D	S2-12D	S2-21D	S2-22D	S2-31D	S2-32D
6th Ave./Exit 260	0.00	0.00	0.00	0.00	0.00	0.00
3rd Ave./Exit 260B	0.00	0.00	0.00	0.00	0.00	0.00
4th Ave./Exit 259B	0.00	0.00	0.00	0.00	0.00	0.00
6th Ave./Exit 259A	0.00	0.00	0.00	0.00	0.00	0.00
University Blvd./Exit 259	0.00	0.00	0.00	0.00	0.00	0.00
Green Springs Ave./Exit 258	0.00	0.00	0.00	0.00	0.00	0.00
Oxmoor Rd./Exit 256	0.00	0.00	0.00	0.00	0.00	0.00
Lakeshore Dr./Exit 255	0.00	**0.19**	0.00	0.00	0.00	0.00
Alford Ave./Exit 254	**0.02**	**2.80**	0.00	**1.79**	**0.01**	**2.87**
US-31/Exit 252 (incident)	**0.02**	**0.43**	**0.02**	**0.55**	**0.03**	**0.63**
I-459/Exit 250	0.00	0.00	0.00	0.00	0.00	0.00
Valleydale Rd./Exit 247	0.00	0.00	0.00	0.00	0.00	0.00

When drivers are informed about the incident presence, they have the opportunity to choose alternate routes that would optimize their travel. As a result, the average additional delay experienced by a driver that traverses the entire study corridor in S2-12D is 3.42 min as compared to 7.7 min in S2-12S. Note that the difference in delays is due to the information availability as the number of lanes closed (2 lanes) and closure duration (1 hour) are both similar in those two scenarios. The delay savings in the scenarios considering information provision are very impressive and become even more notable as the duration of the lane closure increases (3.5 min additional delay in S2-32D compared to 14.75 min in S2-32S). Such comparisons further support the conclusion that the negative effects of an incident on traffic operations can be reduced if drivers are aware of the incident and willing to reroute so as to optimize their travel.

### 3.3. Secondary Incident Scenario Results—Scenario 3

As stated earlier, in *Scenario 3 (S3)*, the primary incident conditions were assumed the same as in S2-22 (*i.e.*, two lanes closed for 2 hours), however, a secondary incident was introduced along the responding EMS response unit route to the main hospital resulting in a two-lane blockage at the I-65 N/Lakeshore Dr. junction. The secondary incident blockage started at 4:20 PM and lasted for 30 min.

**Figure 3:**
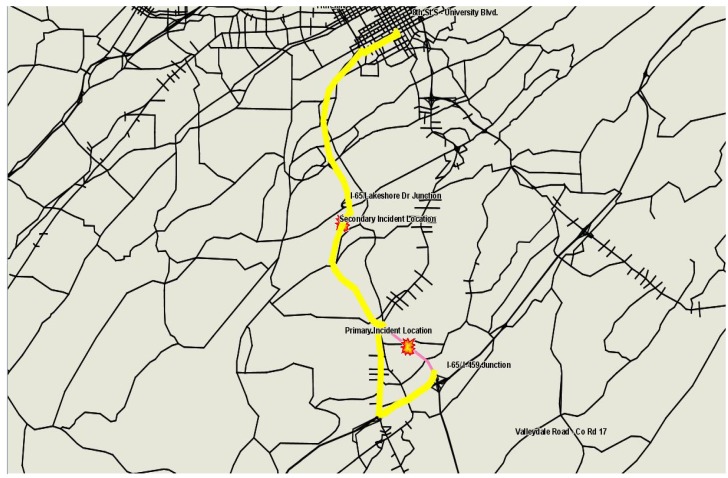
EMS vehicle’s path to UAB hospital in S3-22S.

**Figure 4:**
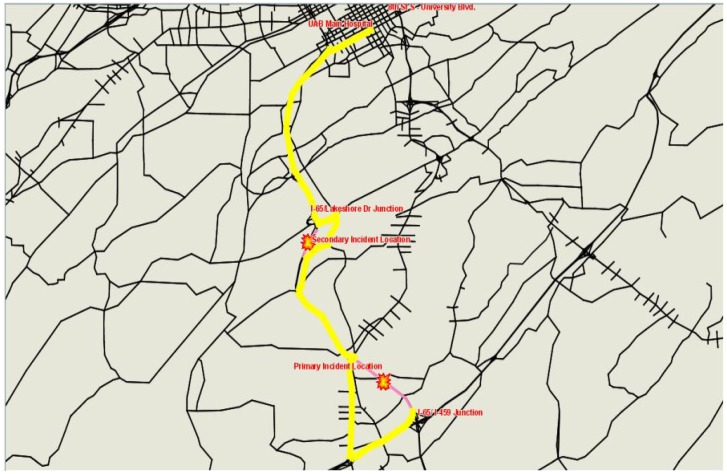
EMS vehicle’s path to UAB hospital with diversion in S3-22D.

Assuming an incident notification, verification, and dispatching time of 5 min, the EMS vehicle arrived at the scene at 4:14 PM and departed en route to UAB Children’s Hospital with an injured person on 4:35 PM. Scenario S3-22S assumed that the EMS driver followed his original best path to the hospital (Figure 3) while S3-22D assumed that the EMS driver was given information and was rerouted around the secondary incident to avoid congestion (Figure 4).

The results summarized in Table 11 show that the occurrence of a secondary incident along the route of the EMS response vehicle to the hospital resulted in a significant increase in travel time (as compared to S2-22S). This is due to the location, severity, and timing of the secondary incident. When the EMS driver followed a diversion, a delay savings of over 2 min was realized.

**Table 11:** EMS Vehicle Travel Times to the Hospital.

Scenario Name	Travel Time (sec)	Travel Time (min)	Information Provision	Secondary Incident
**S2-22S**	1,242	20.7	N	N
**S3-22S**	2,322	38.7	N	Y
**S3-22D**	2,196	36.6	Y	Y

It should be noted that the scenarios and findings presented above are illustrative of the types of analyses and outputs that one can expect by running various incident scenarios on the VISTA Birmingham test bed. While different incident scenario assumptions are expected to lead to different results, the extensive model testing undertaken in this study demonstrates the model capabilities, confirms that model validity and realism, and allows for follow up studies in support of incident and emergency management in the future.

## 4. Conclusions

Successful traffic incident management programs depend on strong interagency involvement and commitment. To meet the safety and mobility needs of all affected parties, traffic incidents require a high level of collaboration and coordination [5]. Furthermore, consideration of actual traffic conditions in the event of an incident plays an important role in minimizing the incident impact on network operations and emergency response.

Dynamic Traffic Assignment (DTA) models are capable of capturing dynamically changing traffic conditions, and thus are superior to more widely used static models for incident and emergency management applications. Using the Visual Interactive System for Transport Algorithms (VISTA) platform, a recently emerged DTA simulation and optimization model, this study investigated the impact of incidents of varying severity and duration on transportation network performance in the Birmingham area. The intensity and extent of the impact over space and time were assessed on the basis of average speeds. Moreover, delays and travel times were considered in order to gain a complete picture of incident-induced impacts on traffic operations and emergency response. Detailed models were developed to capture driver route choices in the event of incident information provision (or the lack of). Moreover, first responders travel times to the scene of the incident were collected to identify best units for responding of the incident, in an effort to improve current dispatching practices. Finally, a secondary incident on the EMS to the hospital was considered to further demonstrate the superiority of Dynamic Traffic Assignment (DTA) over traditional static assignment methods in capturing dynamically changing traffic conditions. 

One of the contributions of this work is that it shows how a mesoscopic DTA model can be used to assist decision making for incident management and emergency response at the regional level. Given the limited studies of this nature, details are offered on simulation-model selection, data collection, model development, assumptions made, and scenario development and testing.

The development of the VISTA prototype model for the Birmingham region involved extensive data collection and processing, customized data coding, and model refinement. More than 1.8 million trips with more than 35 million route options were simulated for each afternoon peak period starting from 4:00 PM to 7:00 PM for each run performed for each of the study scenarios. The availability of the model eliminates the need for repetition of this tedious process in the future, a significant benefit from this effort. Since the model is accessible through the internet it can become available and used beyond the scope of this study by a variety of users for future testing and evaluation studies, with minimum requirements for data collection and coding.

The results of the incident case studies considered in this work demonstrate clearly that the number of lane closures in the event of an incident have greater and longer lasting impacts on network operations than the duration of the lane closures. 

Under the study assumptions, a 1-lane closure had minimal impact on average speeds and delays as compared to normal traffic conditions. This implies that the incident link and upstream links have enough reserve capacity to absorb the changes in the traffic demand during the 1-lane closure without noticeable degradation of the link and corridor performance. On the other hand, lane closures of two lanes had significant impacts on traffic operations, that extended further into space and time as the duration of the two lane closure increased from 1 to 2 or 3 hours. For example, compared to the base case, a 1-hour long 2-lane closure due to an incident (S2-12S) introduced 7.7 min of extra delay to the average driver that traversed the study corridor, while a 2-hour 2-lane closure resulted in 14.75 min of non-recurrent delay. 

Congestion spillback and time for traffic recovery were also significantly impacted when a 2-lane close occurred. It should be noted that the impact of the incident on traffic operations extended over five links (nearly 6 miles) and intensified when the 2 lane closure lasted for 2 hours (S2-22S) instead of one (S2-12S) in which case the incident affected only 3 links. However, extension of the incident duration to 3 hours had only minor incremental effects on speed reduction compared to that observed under the two hours incident assumption.

Furthermore, the results from the parametric analysis clearly demonstrated the impact of incident information dissemination on incident response and recovery. The findings confirm that the negative effects of an incident on traffic operations can be reduced noticeably if drivers are aware of the incident and willing to reroute so as to optimize their travel. Significant reductions in both the severity and extent of congestion were observed in scenarios that considered information provision (S2-S) when compared to their counterparts considering uninformed travelers (S2-D). More specifically, the delay savings in the scenarios considering information availability become more notable as the number and the duration of lane closures increased (from 3.5 min additional delay in S2-32D to 14.75 min in S2-32S). These findings stress the importance of Intelligent Transportation Systems (ITS) technologies for the collection and dissemination of information to the public during incidents and emergencies. ITS applications provide effective ways to collect information on road and traffic conditions and to deliver information to the public in a timely manner, thus assisting in incident management. An assessment of existing ITS capabilities and needs around incident prone locations in the Birmingham area is recommended in an effort to improve incident management in the future.

Last but not least, the case study results allowed for selection of the best units to respond to the incident, as well as best routes to the incident site and from the incident site to the hospital location taking under consideration actual network traffic conditions during the incident presence. This model capability allows for improved dispatching decisions that in turn can improve incident response and recovery operations.

It should be noted that in this study the developed model performed only off-line analyses. Ultimately, the model should be expanded to support decision making during the course of an incident event in real time. More specifically, a module should be designed in VISTA that will (a) allow incorporation of real-time data into the model and (b) be capable of running faster than real time. Using such a module for incident management, the effects from dynamic events on the roadway capacity and driver behaviors could be emulated and optimized solutions can be obtained to minimize on traffic operations and emergency response.
